# Template for documenting and reporting data in physician-staffed pre-hospital services: a consensus-based update

**DOI:** 10.1186/s13049-020-0716-1

**Published:** 2020-04-03

**Authors:** Kristin Tønsager, Andreas Jørstad Krüger, Kjetil Gorseth Ringdal, Marius Rehn, the P-EMS Template Collaborating Group, Bjørn Hossfeld, Ivo Breitenmoser, Mohyudin Dingle, Attila Eröss, Francisco Gallego, Peter Hilbert-Carius, Jo Kramer-Johansen, Jouni Kurola, Leif Rognås, Patrick Schober, Ákos Soti

**Affiliations:** 1 Department of Research, The Norwegian Air Ambulance Foundation, Post box 414, Sentrum, N-0103, Oslo, Norway; 2 Department of Anaesthesiology and Intensive Care, Stavanger University Hospital, Stavanger, Norway; 3 Faculty of Health Sciences, University of Stavanger, Stavanger, Norway; 4 Department of Emergency Medicine and Pre-Hospital Services, St. Olavs Hospital, Trondheim, Norway; 5 Department of Anaesthesiology, Vestfold, Hospital Trust, Tønsberg, Norway; 6 Norwegian Trauma Registry, Oslo University Hospital, Oslo, Norway; 7 Pre-hospital Division, Air Ambulance Department, Oslo University Hospital, Oslo, Norway

**Keywords:** Documentation, Data collection, Pre-hospital, Physician, Emergency medical services, Consensus, Air ambulances, Quality of health care

## Abstract

**Background:**

Physician-staffed emergency medical services (p-EMS) are resource demanding, and research is needed to evaluate any potential effects of p-EMS. Templates, designed through expert agreement, are valuable and feasible, but they need to be updated on a regular basis due to developments in available equipment and treatment options. In 2011, a consensus-based template documenting and reporting data in p-EMS was published. We aimed to revise and update the template for documenting and reporting in p-EMS.

**Methods:**

A Delphi method was applied to achieve a consensus from a panel of selected European experts. The experts were blinded to each other until a consensus was reached, and all responses were anonymized. The experts were asked to propose variables within five predefined sections. There was also an optional sixth section for variables that did not fit into the pre-defined sections. Experts were asked to review and rate all variables from 1 (totally disagree) to 5 (totally agree) based on relevance, and consensus was defined as variables rated ≥4 by more than 70% of the experts.

**Results:**

Eleven experts participated. The experts generated 194 unique variables in the first round. After five rounds, a consensus was reached. The updated dataset was an expanded version of the original dataset and the template was expanded from 45 to 73 main variables. The experts approved the final version of the template.

**Conclusions:**

Using a Delphi method, we have updated the template for documenting and reporting in p-EMS. We recommend implementing the dataset for standard reporting in p-EMS.

## Background

Physician-staffed emergency medical services (p-EMS) are common in European countries, and they provide highly specialized, goal-directed therapy. Pre-hospital physicians have the potential to restore adequate flow and physiology in severely sick or injured patients, but the subject remains debated [1–6]. P-EMS are resource demanding compared with standard paramedic-staffed services [7], and more research is needed to evaluate any potential effects of p-EMS [1, 8, 9]. High-quality research relies on data quality and uniform documentation is essential to ensure reliable and valid data. Currently, p-EMS data are low quality, and the lack of systematic documentation complicates comparison, creating a barrier for high-quality outcome research [10].

In 2011, a consensus-based template for documenting and reporting data in p-EMS was published [7]. Templates for uniform documentation may facilitate international multi-centre studies, thereby increasing the quality of evidence [11]. Such templates, designed through expert agreement, are valuable and feasible, but they need to be updated on a regular basis due to developments in available equipment and treatment options [12–15]. The p-EMS template has been incorporated for daily use in Finland, but it has not yet been implemented in other European countries. A recent study concluded that the published template is feasible for use in p-EMS and that a large amount of data may be captured, facilitating collaborative research [16]. However, the feasibility study revealed areas for improvement of the template. To make the template even more relevant, further revisions should be made.

The aim of this study was to revise and update the template for documenting and reporting in p-EMS through expert consensus [7] using the Delphi method.

## Methods

### The experts

No exact criterion exists concerning selection of participants for a Delphi study.

Many European countries share similarities with regards to infrastructure, socio-political system and health care services, favouring research collaboration [17]. Representatives from European p-EMS were invited to join an expert panel using the same inclusion criteria as the original template:

1. Clinical experience by working in p-EMS to ensure personal insight into the operative and medical characteristics of advanced pre-hospital care.
2. Scientific and/or substantial leadership responsibilities in pre-hospital care to ensure competency in research methods and governance of pre-hospital emergency systems.
3. Ability to communicate in English.

The experts were identified via the European Prehospital Research Alliance (EUPHOREA) network. The EUPHOREA network consists of representatives from p-EMS throughout central Europe, UK and Scandinavia. Experts were invited via e-mail. Non-responders were reminded via e-mail. For all rounds non-responders were reminded twice per e-mail.

### The Delphi method

A Delphi technique was applied to achieve a consensus from a panel of selected experts interacting via e-mail. No physical meetings were held. A research coordinator interacted with the participants, administered questionnaires and collected the responses until a consensus was reached. The experts were blinded to each other until an agreement was reached. All responses were anonymized. The Delphi process ran from Feb. 19 to Oct. 1, 2019. The final dataset was approved by all experts.

### Objectives for each round of the Delphi process

The experts were asked to propose variables within each of five predefined sections:

1. **Fixed system variables**
  Variables describing how the p-EMS is organized, competence in the p-EMS team and its operational capacities (e.g., dispatch criteria, population, mission case-mix and equipment utilized by the services). These data do not change between missions and are considered fixed.
2. **Event operational descriptors**
  Variables documenting the mission context (e.g., data on logistics, type of dispatch, time variables and mission type).
3. **Patient descriptors**
  Variables documenting patient state (e.g., age, gender, comorbidity, patient physiology and medical complaint).
4. **Process mapping variables**
  Variables documenting diagnostic and therapeutic procedures (e.g., monitoring, medication, airway devices used, etc.) performed during the period of p-EMS care.
5. **Outcome and quality indicators**
  Variables describing patient outcome and quality.

There was also an optional sixth section for proposals of variables that did not fit into one of the pre-defined sections.

### Round I

Each expert suggested 10 variables considered to be most important for routine documentation in p-EMS within each of the five predefined sections.

### Round II

The results from the first round were structured in a worksheet (Excel for Mac, version 16.31, 2019 Microsoft). Duplicate suggestions were removed before the variables were returned to the experts. Variables from the original template were included if not suggested by the experts. Experts were asked to review and rate all variables from 1 (totally disagree) to 5 (totally agree) based on relevance.

### Round III

Variables rated ≥4 by more than 70% of the experts were included in the template draft and presented to the experts [18, 19]. In addition, the experts received a number of questions pertaining to the wording of questions, consent to delete some questions because of overlap, relevance of alternatives under a main question, and whether there should be a free-text field for addressing key lessons. Furthermore, they were instructed to provide comments and grade the variables as either compulsory or optional. Later, the experts were asked to suggest the frequency of variable reporting (for each mission, monthly or annually). Variables rated ≥4 by less than 50% of the experts were excluded. Variables rated ≥4 by more than 50% of the experts were summarized and re-rated by the experts. If more than 70% of the experts rated a variable ≥4 in this second round, the variable was included in the final template.

### Round IV

After summarizing the feedback from round III, the list of variables achieving consensus, accompanying comments, and further questions were distributed to the experts. All variables were numbered. This round provided an opportunity for the experts to revise their judgements and combine similar variables.

### Round V

Feedback from round IV was summarized into a final version of the template and sent to the experts to elicit any objections and/or to give final approval of the template for routine reporting in p-EMS.

The study was drafted according to the Standards for Reporting Qualitative Research (SRQR) [20].

## Results

### The experts

Thirty experts were invited to join the consensus process and 15 agreed to participate. Eleven experts responded in the first Delphi round, ten responded in the second round and nine responded in the last three rounds.

### Round I

**Fig. 1:**
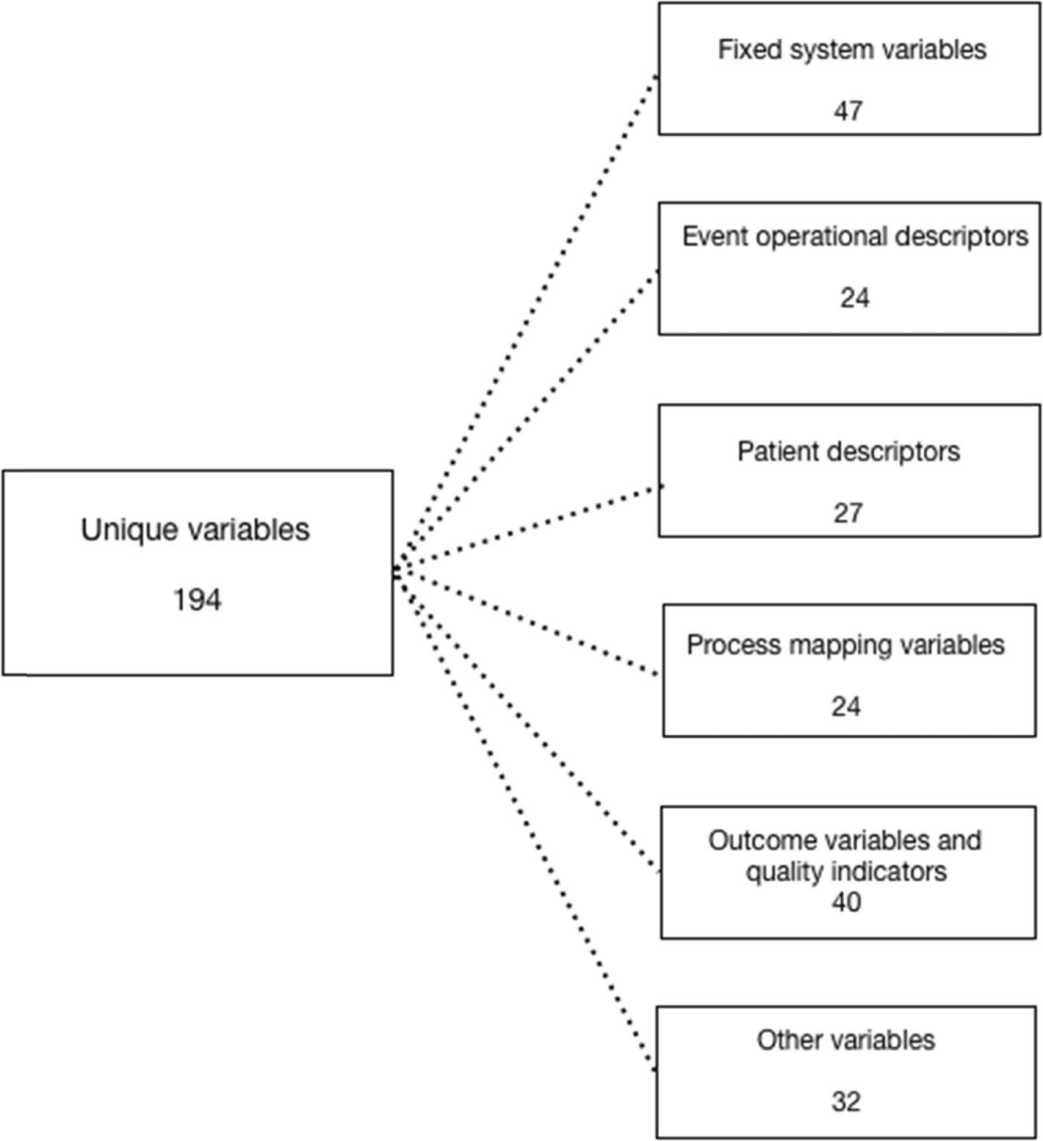
Suggested variables. Number of suggested variables for the different sections in the first round of the Delphi process

The experts suggested 194 unique variables in the first round (Fig. 1). All variables from the original template were among the suggested variables.

**Table 1:** Fixed system variables

Data variable number	Main data variable name	Type of data	Data variable categories or values	Data variable sub-categories or values	Type	Definition of data variable	How often should variable be reported
**1. Fixed system variables**							
**1.1.**	Specialty of physicians	Categorical	1.1.1 Anaesthesiology		Check box	Specialty of physicians working in the service on a regular basis	Annual
			1.1.2 Emergency medicine				
			1.1.3 Intensive care				
			1.1.4 Surgery				
			1.1.5 Internal medicine				
			1.1.6 Other				
**1.2.**	Training level of physicians	Categorical	1.2.1 Trainee/registrar		Check box		Annual
			1.2.2 Specialist				
**1.3.**	Composition of team	Categorical	1.3.1 Nurse		Check box	Qualification of non-p-EMS personnel accompanying the physician during mission	Annual
			1.3.2. Paramedic			As defined by each national service	
			1.3.3. EMS-technician			As defined by each national service	
			1.3.4. Other				
**1.4.**	Catchment population	Continuous			Number	Number of citizens in the area covered by the service on a regular basis	Annual
**1.5.**	Catchment area	Continuous	1.5.1. Square km		Number	Area in which the service is planned to operate on a regular basis, square km	Annual
		Categorical	1.5.2. Type	1.5.2.1. Urban	Check box	Type of area where service operate on a regular basis (as defined by each service)	
				1.5.2.2. Rural			
**1.6.**	Does the service conduct primary missions?	Categorical	1.6.1. Yes		Bullet list	On-scene missions	Annual
			1.6.2. No				
**1.7.**	Does the service conduct inter-hospital transfer missions?	Categorical	1.7.1. Yes		Bullet list	Patient transfers between different hospitals or facilities	Annual
			1.7.2. No				
**1.8.**	Number of consultations only (advice) per year	Continuous			Number	Physician is consulted by EMS or other professionals (give advice)	Annual
**1.9.**	Number of primary missions per year	Continuous			Number	Missions where physician is on-scene. Total number for the service	Annual
**1.10.**	Number of inter-hospital transfer missions per year	Continuous			Number	Inter-hospital or interfacility transfer. Total number for the service	Annual
**1.11.**	Number of cancelled missions per year	Continuous			Number	Any mission where p-EMS is alarmed but not able to respond or must interrupt mission	Annual
**1.12.**	Number of events per year per physician	Continuous			Number	The average number of missions per individual physician per year	Annual
**1.13.**	Number of events for p-EMS unit/100,000 inhabitants per year	Continuous			Number		Annual
**1.14.**	Number of EMS events/100,000 inhabitants per year	Continuous			Number	Number of events for the whole EMS system, including p-EMS	Annual
**1.15.**	Number of p-EMS units/ 100,000 inhabitants	Continuous			Number		Annual
**1.16.**	Number of p-EMS units/km2	Continuous			Number	Area in which the service operates on a regular basis	Annual
**1.17.**	Available vehicles in service	Categorical			Check box	Available vehicles on a regular basis for p-EMS	Annual
			1.17.1. Rapid response car			Regular car, no stretcher	
			1.17.2. Regular ambulance staffed with physician			Car with stretcher. Physician is attending on a regular basis	
			1.17.3. Rotor Wing				
			1.17.4. Fixed Wing				
			1.17.5. Boat staffed with physician			Physician is attending on a regular basis	
			1.17.6. Other				
**1.18.**	Operating hours	Categorical	1.18.1. Daytime		Bullet list	Regular working hours, e.g., 08–16, as defined by each service	Annual
			1.18.2. Daylight only			Service operates only in daylight (different opening hours during the year due to seasonal variations). Daylight as defined by each service	
			1.18.3. 24/7 (full-time service)			Service operates during the day and night	
			1.18.4. Other				
**1.19.**	Activation criteria	Categorical	1.19.1. Criteria based		Check box	P-EMS activated in accordance with a pre-defined set of activation criteria used by EMCC	Annual
			1.19.2. Consultation with physician			Physician-staffed unit activated only after consultation with an on-call physician	
			1.19.3. Individual			No predefined criteria for activation of p-EMS	
**1.20.**	Dispatch system	Categorical	1.20.1. Integrated EMCC		Check box	Integrated EMCC includes dispatch centres coordinating all levels of pre-hospital services	Annual
			1.20.2. Special EMCC			Special EMCC includes centres only responsible for p-EMS units	
			1.20.3. Other				
**1.21.**	Advanced equipment carried by service	Categorical	1.21.1. Blood products		Check box	Advanced equipment available on a regular basis to service	Annual
			1.21.2. Mechanical chest compression device				
			1.21.3. Ultrasound				
			1.21.4. Advanced drugs			Drugs not available to regular EMS in the individual system	
			1.21.5. Additional airway management equipment (e.g., videoscope)			Airway management equipment beyond the scope of regular EMS	
			1.21.6. Surgical procedures supported			Service carries equipment for predefined surgical procedures	
**1.22.**	Does a system for registration and reviewing of adverse events, critical incidents and educational events in the service exist?	Categorical	1.22.1. Yes		Bullet list		Annual
			1.22.2 No				
**1.23.**	Categorization of events/case mix	Categorical	1.23.1. Cardiac arrest medical aetiology		Check box	Mission types the service responds to	Annual
			1.23.2. Cardiac arrest traumatic aetiology				
			1.23.3. Trauma				
			1.23.4. Breathing difficulties				
			1.23.5. Myocardial infarction (MI)			Confirmed by ECG	
			1.23.6. Chest pain, MI not confirmed				
			1.23.7. Stroke				
			1.23.8. Acute neurology excluding stroke				
			1.23.9. Reduced level of consciousness				
			1.23.10. Poisoning/Intoxication				
			1.23.11. Burns				
			1.23.12. Obstetrics and childbirth				
			1.23.13. Infection				
			1.23.14. Anaphylaxis				
			1.23.15. Surgical				
			1.23.16. Asphyxiation				
			1.23.17. Drowning				
			1.23.18. Psychiatry excluding poisoning/intoxication				
			1.23.19. All of the above			Service responds to all types of events	
			1.23.20. Other				
**1.24.**	Number of intubations successful on first attempt and without desaturation (DASH1a intubations) /100 intubations	Continuous			Number		Annual
**1.25.**	Number of patients where blood glucose was measured after ROSC/100 ROSC	Continuous			Number		Annual

EMS- Emergency medical services, p-EMS - Physician-staffed emergency medical services, EMCC - Emergency medical communication centre, MI - Myocardial infarction, ECG – Electrocardiogram, DASH1a - Definitive airway sans hypoxia/hypotension on first attempt,

ROSC – Return of spontaneous circulation

### Round II

The experts rated the variables suggested in round I from 1 (totally disagree) to 5 (totally agree) based on relevance. A total of 68 main variables (24 fixed system variables, 10 event operational descriptors, 15 patient descriptors, 10 process mapping variables, 9 outcome and quality indicators and no other variables) were rated ≥4 by more than 70% of the experts and included in the preliminary template. Thirty-five main variables and 32 sub-variables were rated < 4 by 50–70% of the experts. Ninety-one variables were rated ≥4 by less than 50% of the experts and were excluded.

### Round III

**Fig. 2:**
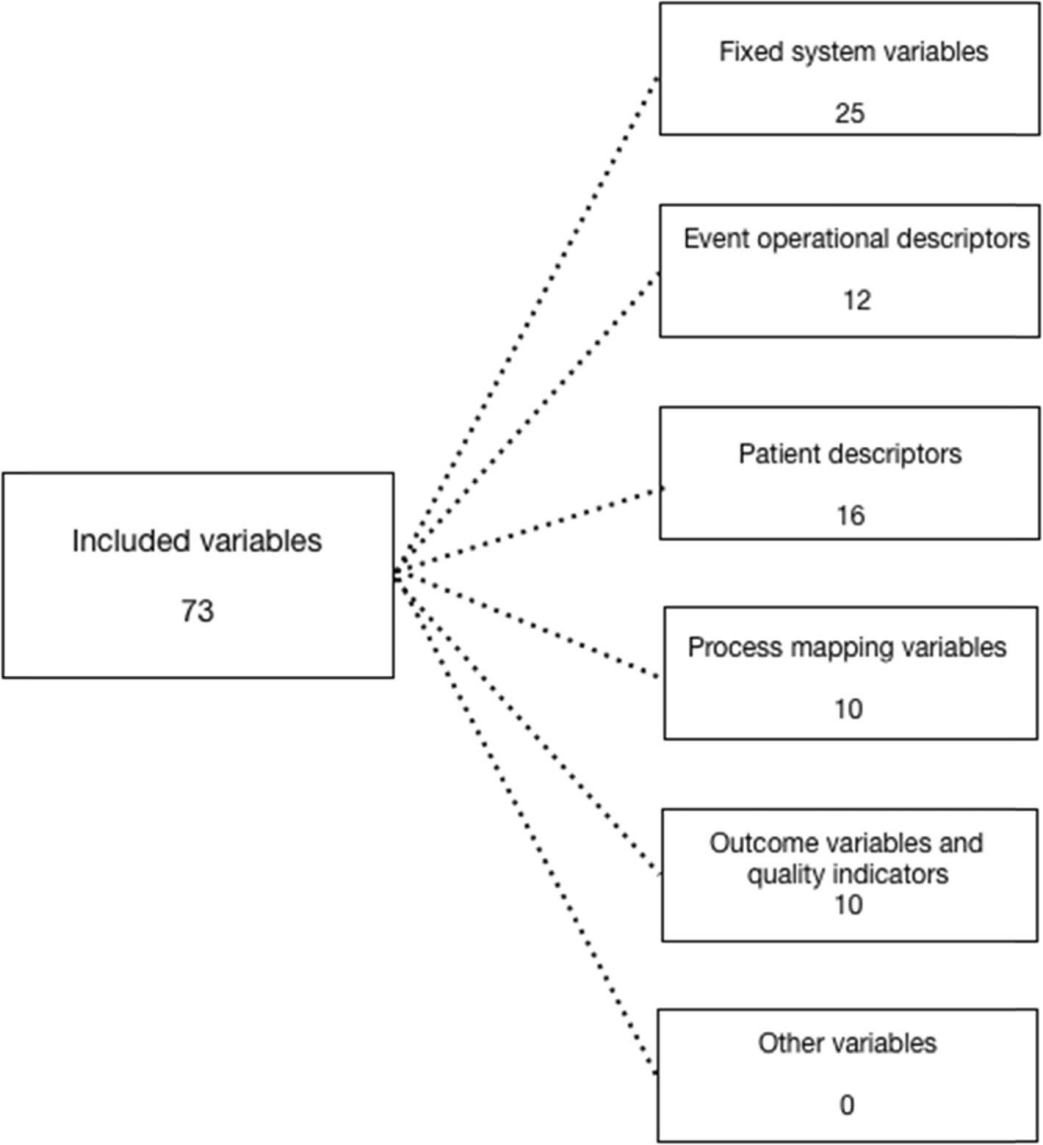
Included variables. Final number of variables included in the updated template

The preliminary template was presented to the experts. Additionally, the experts rated the 35 main variables and 32 sub-variables that were initially rated ≥4 by 50–70% once more. Five more main variables and 9 sub-variables were included after this second rating. In total, 73 main variables were included (Fig. 2). The experts agreed that all fixed system variables should be reported annually while all event operational descriptors, patient descriptors, process mapping variables and outcome and quality indicators should be reported after each mission.

**Table 2:** Event operational descriptors

Data variable number	Main data variable name	Type of data	Data variable categories or values	Data variable sub-categories or values	Type	Definition of data variable	How often should variable be reported
**2. Event operational descriptors**							
2.1.	Time points	Continuous					For each mission
			2.1.1. Call received at EMCC		hh:mm	When the alarm call is answered at the initial EMCC	
			2.1.2. Time of system activation (dispatch time)		hh:mm	When EMCC dispatch p-EMS	
			2.1.3. Unit en route/take-off time		hh:mm	When vehicle starts to move (car or rotor wing/fixed wing)	
			2.1.4. Unit arrival on scene		hh:mm	When vehicle stops at a location as close as possible to the patient	
			2.1.5. Time of first physician contact with patient		hh:mm	When pre-hospital physician arrives at patient site	
			2.1.6. Time when patient leaves scene		hh:mm	When patient is transferred from the original location or time of death if dead on scene	
			2.1.7. Time when patient arrives at hospital (or alternative site if not delivered to hospital)		hh:mm	When the patient is formally transferred to receiving medical facility personnel	
2.2.	Date of event	Continuous			dd.mm.yyyy	The date the unit was dispatched	For each mission
2.3.	Type of mission/dispatch	Categorical			Check box		For each mission
			2.3.1. Primary medical mission			Includes all primary missions other than trauma (medical, surgical, paediatric, obstetric)	
			2.3.2. Primary trauma mission			Includes all primary trauma missions	
			2.3.3. Inter-hospital transfer mission			Inter-hospital or inter-facility mission	
			2.3.4. SAR mission				
			2.3.5. Major incident response				
			2.3.6. Contingency				
			2.3.7. Rendezvous with ambulance				
			2.3.8. Consultation				
			2.3.9. Single patient			Only one patient treated by p-EMS during the mission	
			2.3.10. Multiple patients			More than one patient treated by p-EMS during the mission	
			2.3.11. Other				
2.4.	Dispatch criteria	Categorical			Check box	Medical reason for dispatch	For each mission
			2.4.1. Medical				
			2.4.2. Trauma				
			2.4.3. Neurologic				
			2.4.4. Obstetric				
			2.4.5. Burn				
			2.4.6. Other				
2.5.	Activation type	Categorical			Bullet list		For each mission
			2.5.1. Primary mission				
				2.5.1.1. Initiated by dispatch centre			
				2.5.1.2. Requested dispatch from other units			
				2.5.1.3. Other			
		Categorical	2.5.2. Inter-hospital transfer mission				
				2.5.2.1. Physician-staffed unit used because of level of treatment during transport			
				2.5.2.2. Physician-staffed unit used because of speed of transport			
				2.5.2.3. Both above			
				2.5.2.4. Other			
2.6.	Mode of transportation to scene	Categorical			Bullet list	Main type of vehicle used to get p-EMS to the scene	For each mission
			2.6.1. Rapid response car			Regular car, no stretcher	
			2.6.2. Regular ambulance			Car with stretcher	
			2.6.3. Rotor Wing				
			2.6.4. Fixed Wing				
			2.6.5. Boat staffed with physician				
			2.6.6. Other				
2.7.	Mode of transportation from scene	Categorical			Bullet list	Main type of vehicle used to transport the patient to definitive care	For each mission
			2.7.1. Rapid response car				
			2.7.2. Regular ambulance staffed with physician			Physician is part of the ambulance crew on a regular basis	
			2.7.3. Regular ambulance with physician attending			Ambulance crew normally without a physician, physician is attending because of patient need	
			2.7.4. Patient transported in ambulance without physician				
			2.7.5. Rotor wing				
			2.7.6. Fixed wing				
			2.7.7. Boat staffed with physician				
			2.7.8. Patient not transported due to no indication				
			2.7.9. Patient not transported due to patient refusal				
			2.7.10. Patient dead and not transported				
			2.7.11. Other				
2.8.	Result of dispatch				Bullet list	Dispatch means unit alarmed for mission or request/advice/supervision	For each mission
		Categorical	2.8.1. Patient attended			P-EMS attended the patient	
				2.8.1.1. Transported with physician escort			
				2.8.1.2. Transported without physician escort			
				2.8.1.3. Discharged on-scene		Patient not transported	
				2.8.1.4. Pronounced dead on scene			
		Categorical	2.8.2. Patient not attended			P-EMS did not attend the patient. The main reason why mission is aborted or refused	For each mission
				2.8.2.1. Weather			
				2.8.2.2. Technical reasons			
				2.8.2.3. Other mission (concurrency)			
				2.8.2.4. Alternative tasking			
				2.8.2.5. Mission refused due to duty time limitations			
				2.8.2.6. Fatigue			
				2.8.2.7. Not needed			
				2.8.2.8. No time benefit			
				2.8.2.9. Patient has left scene (before arrival of unit)			
				2.8.2.10. Mission taken over by another p-EMS			
2.9.	Trauma mechanism	Categorical			Bullet list	The Utstein Trauma template. The mechanism (or external factor) that caused the injury event	For each mission
			2.9.1. Not trauma				
			2.9.2. Blunt trauma				
				2.9.2.1. Traffic - motor vehicle injury		Car, pickup, truck, van, heavy transport vehicle, bus	
				2.9.2.2. Traffic - motorbike			
				2.9.2.3. Traffic - bicycle			
				2.9.2.4. Traffic - pedestrian			
				2.9.2.5. Traffic - other		Ship, airplane, railway train	
				2.9.2.6. Fall from same level		Low energy fall. From the persons height or less	
				2.9.2.7. Fall from higher level		High energy fall. From more than the persons height	
				2.9.2.8. Struck or hit by blunt object		Tree, tree branch, bar, stone, human body part, metal, other	
				2.9.2.9. Explosives		Blast injuries	
				2.9.2.10 Other			
				2.9.2.11. Unknown			
			2.9.3. Penetrating trauma				
				2.9.3.1. Stabbed by pointed or sharp object		Knife, sword, dagger or other	
				2.9.3.2. Gun		By handgun, shotgun, rifle, or another firearm of any dimension	
				2.9.3.3. Other			
			2.9.4. Unknown			Unknown trauma mechanism	
2.10.	Specialty of the attending physician	Categorical			Check box	The pre-hospital physician attending patient on scene	For each mission
			2.10.1. Anaesthesiology				
			2.10.2. Emergency medicine				
			2.10.3. Intensive care				
			2.10.4. Surgery				
			2.10.5. Internal medicine				
			2.10.6. Other				
2.11.	NACA score	Categorical			Bullet list	NACA 0–7	For each mission
			2.11.1. NACA 0				
			2.11.2. NACA 1				
			2.11.3. NACA 2				
			2.11.4. NACA 3				
			2.11.5. NACA 4				
			2.11.6. NACA 5				
			2.11.7. NACA 6				
			2.11.8. NACA 7				
			2.11.9. NACA score unknown				
2.12.	Where patient is delivered	Categorical			Bullet list	Where physician-staffed unit delivers patient	For each mission
			2.12.1. Major Trauma Centre/Definitive care centre			Hospital where all definitive treatment is available (to the particular patient)	
			2.12.2. Local hospital			Hospital where all definitive treatment is not available (to the particular patient)	
			2.12.3. Other health care facility			Facility not defined as hospital	

*EMCC* Emergency medical communication centre, *SAR* Search and rescue, *p-EMS* physician-staffed emergency medical services, *NACA score* National Advisory Committee for Aeronautics score

**Table 3:** Patient descriptors

Data variable number	Main data variable name	Type of data	Data variable categories or values	Data variable sub-categories or values	Type	Definition of data variable	How often should variable be reported
**3. Patient descriptors**							
**3.1.**	Age	Continuous			Number	Patient age at the time of event	For each mission
**3.2.**	Gender	Categorical	3.2.1 Female		Bullet list	Patient gender	For each mission
			3.2.2 Male				
			3.2.3 Unknown				
**3.3.**	Pre-event comorbidity	Ordinal			Bullet list	Pre-event ASA-PS. The comorbidity existing before event. Derangements from present disease should not be considered	For each mission
			3.3.1 ASA-PS 1			A normal healthy patient	
			3.3.2 ASA-PS 2			A patient with mild systemic disease	
			3.3.3 ASA-PS 3			A patient with severe systemic disease	
			3.3.4 ASA-PS 4			A patient with severe systemic disease that is a constant threat to life	
			3.3.5 ASA-PS 5			A moribund patient who is not expected to survive without operation	
			3.3.6 ASA-PS 6			A declared brain-dead patient whose organs are being removed for donor purposes	
			3.3.7 ASA Unknown				
**3.4.**	Chronic medications	Categorical	3.4.1 Yes		Bullet list	Does patient use medication on a regular basis?	For each mission
			3.4.2 No				
			3.4.3 Unknown				
**3.5.**	Medical problem	Categorical			Bullet list	The condition most likely to be the patient’s true medical problem, main clinical symptom or diagnosis, decided by attending p-EMS	For each mission
			3.5.1 Cardiac arrest medical aetiology				
			3.5.2 Cardiac arrest traumatic aetiology				
			3.5.3 Trauma				
			3.5.4 Breathing difficulties				
			3.5.5 Myocardial infarction (MI)			Confirmed by ECG	
			3.5.6 Chest pain, MI not confirmed				
			3.5.7 Stroke				
			3.5.8 Acute neurology excluding stroke				
			3.5.9. Reduced level of consciousness			Aetiology unknown	
			3.5.10 Poisoning/Intoxication				
			3.5.11 Burns				
			3.5.12 Obstetrics and childbirth				
			3.5.13 Infection				
			3.5.14 Anaphylaxis				
			3.5.15 Surgical				
			3.5.16 Asphyxiation				
			3.5.17 Drowning				
			3.5.18 Psychiatry excluding poisoning/intoxication				
			3.5.19 Other				
**3.6.**	Glasgow Coma Scale	Ordinal	3.6.1 First		Number	First recorded pre-interventional GCS upon arrival of p-EMS	For each mission
			3.6.2 Last			GCS at end of patient care or patient handover	
			3.6.3 Not recorded				
		Categorical	3.6.4 Patient intubated	3.6.4.1 Yes	Bullet list		
				3.6.4.2 No			
**3.7.**	Heart rate	Continuous			Number	Documented by ECG (1st choice), palpation or SpO2 curves (3rd choice)	
			3.7.1 First			First heart rate per minute measured by p-EMS upon arrival	
			3.7.2 Last			Heart rate per minute at end of care or patient handover	
			3.7.3 Not recorded				
**3.8.**	Systolic blood pressure	Continuous	3.8.1 Lowest		Number	Lowest recorded systolic blood pressure measured by p-EMS (sphygmomanometer, monitor or intra-arterial line) upon arrival	
			3.8.2 First			First recorded systolic blood pressure measured by p-EMS (sphygmomanometer, monitor or intra-arterial line) upon arrival	
			3.8.3 Last			Systolic blood pressure at end of care or patient handover	
			3.8.4 Not recordable			Not possible to record despite several attempts	
			3.8.5 Not recorded				
**3.9.**	Cardiac rhythm/ECG rhythm	Categorical	3.9.1 First	3.9.1.1 Sinus rhythm	Bullet list	First cardiac rhythm interpreted by p-EMS (minimum 3-channel lead). At primary survey/upon arrival	For each mission
				3.9.1.2. SVES, VESmono			
				3.9.1.3 AF/AFL/AV-block gr II/III, VESpoly			
				3.9.1.4 VF, VT, Asystole, PEA			
				3.9.1.5 Not recorded			
			3.9.2 Last	3.9.2.1 Sinus rhythm		Cardiac rhythm at end of care or patient handover (minimum 3-channel lead)	
				3.9.2.2 SVES, VESmono			
				3.9.2.3 AF/AFL/AV-block gr II/III, VESpoly			
				3.9.2.4 VF, VT, Asystole, PEA			
				3.9.2.5 Not recorded			
**3.10.**	SpO2	Continuous	3.10.1 Lowest		Number	Lowest recorded oxygen saturation by p-EMS (measured with pulse oximeter or arterial blood gas (SaO2))	For each mission
			3.10.2 First			First recorded oxygen saturation by p-EMS (measured with pulse oximeter or arterial blood gas (SaO2) upon arrival)	
			3.10.3 Last			Oxygen saturation at end of care or patient handover	
			3.10.4 Not recordable			Not possible to record despite several attempts	
			3.10.5 Not recorded				
**3.11.**	Oxygen supplementation	Categorical	3.11.1 Oxygen supplementation at first measurement of SpO2	3.11.1.1 Yes	Bullet list	First measurement by p-EMS	For each mission
				3.11.1.2 No			
			3.11.2 Oxygen supplementation at last measurement of SpO2	3.11.2.1 Yes		Last measurement by p-EMS	
				3.11.2.2 No			
**3.12.**	Respiratory rate	Continuous	3.12.1 First		Number	First respiratory rate per minute measured by p-EMS upon arrival. If mechanically ventilated; document ventilation rate	For each mission
			3.12.2 Last			Respiratory rate at end of care or patient handover	
			3.12.3 Not recorded				
**3.13.**	Pain	Categorical	3.13.1 First VAS score		Number	First VAS score assessed by p-EMS upon arrival	For each mission
			3.13.2 Last VAS score			VAS score at end of care or patient handover	
			3.13.3 Not recorded				
**3.14.**	End-tidal CO2	Continuous	3.14.1 First		Number	First end-tidal CO2 measured by p-EMS	For each mission
			3.14.2 Last			Last end-tidal CO2 measured by p-EMS	
			3.14.3 Not recorded				
**3.15.**	Temperature (core)	Continuous	3.15.1 First		Number	First core temperature measured by p-EMS upon arrival	For each mission
			3.15.2 Last			Last core temperature measured by p-EMS	
			3.15.3 Not recorded				
**3.16**	Airway at primary survey	Categorical	3.16.1 Clear		Bullet list	As rated by attending physician	For each mission
			3.16.2 Threatened				
			3.16.3 Obstructed				
			3.16.4 Unknown				

*ASA-PS* American Society of Anesthesiologists physical scale, *p-EMS* physician-staffed emergency medical services, *MI* Myocardial infarction, *ECG* Electrocardiogram, *GCS* Glasgow coma score, *SpO2* Peripheral capillary oxygen saturation, *SVES* Supraventricular extrasystole, *VESmono* Ventricular extrasystole, monomorphic, *AF* Atrial fibrillation, *AFL* Atrial flutter, *AV-block* Atrioventricular block, *VESpoly* Ventricular extrasystole, polymorphic, *VF* Ventricular fibrillation, *VT* Ventricular tachycardia, *PEA* Pulseless electrical activity, *SaO2* Arterial oxygen saturation, *VAS* Visual analogue scale, *CO2* Carbon dioxide

**Table 4:** Process mapping variables

Data variable number	Main data variable name	Type of data	Data variable categories or values	Data variable sub-categories or values	Type	Definition of data variable	How often should variable be reported
**4. Process mapping**							
**4.1.**	Diagnosis and monitoring procedures	Categorical	4.1.1 Blood pressure	4.1.1.1 Non-invasive	Check box	Monitoring used and procedures performed by p-EMS	For each mission
				4.1.1.2 Invasive			
				4.1.1.3. Other			
			4.1.2. SpO2				
			4.1.3. EtCO2			Capnometry or capnography used	
			4.1.4. Temperature (core)			Temperature measured during mission	
			4.1.5. ECG	4.1.5.1. Monitoring (3 or 4-lead or pads)			
				4.1.5.2. Analysis (12-lead)			
			4.1.6. Ultrasound/Doppler	4.1.6.1. FAST		By p-EMS	
				4.1.6.2. Lung for pneumothorax		By p-EMS	
			4.1.7. Point of care (POC) blood gas analysis			By p-EMS	
			4.1.8. Other POC testing			By p-EMS	
			4.1.9. POC lab test			By p-EMS	
			4.1.10. Blood glucose			By p-EMS	
			4.1.11. Other				
			4.1.12. None				
**4.2.**	Drugs used to facilitate airway management	Categorical	4.2.1. Sedatives		Check box	By p-EMS	For each mission
			4.2.2. NMBA				
			4.2.3. Analgesics				
			4.2.4. Local/topic anaesthetics				
			4.2.5. Other				
			4.2.6. None				
**4.3.**	Airway management	Categorical	4.3.1. Oxygen		Check box	Device or procedures used for successful airway management	For each mission
			4.3.2. Manual			Chin-lift, jaw thrust, recovery position	
			4.3.3. Bag Mask Ventilation				
			4.3.4. Nasopharyngeal device				
			4.3.5. Oropharyngeal device				
			4.3.6. SAD 1. generation			Laryngeal mask with no mechanism for protection against aspiration	
			4.3.7. SAD 2. generation			Laryngeal mask with any aspiration protection mechanism	
			4.3.8. Oral ETI				
			4.3.9. Nasal ETI				
			4.3.10. Surgical airway	4.3.10.1. Mac-blade			
				4.3.10.2. Hyper angulated blade			
			4.3.11. Other				
			4.3.12. None				
**4.4.**	Number of attempts to secure airway	Continuous			Number	Number of attempts needed before a definitive airway is in place by p-EMS	For each mission
**4.5.**	Breathing- related procedures	Categorical			Check box	Procedures performed by p-EMS	For each mission
			4.5.1. Controlled manually			Breathing assistance using physician’s hands. Bag valve mask ventilation	
			4.5.2. Controlled mechanically			Use of technical respiratory support; ventilator, NIV	
			4.5.3. Needle decompression				
			4.5.4. Chest tube				
			4.5.5. Thoracostomy				
			4.5.6. Escharotomy				
		Continuous	4.5.7. FiO2			If patient is ventilated	
		Continuous	4.5.8. PEEP			If patient is ventilated	
			4.5.9. Other				
			4.5.10. None				
**4.6.**	Circulation- related procedures	Categorical	4.6.1. Peripheral i.v. line		Check box	Procedures performed by p-EMS	For each mission
			4.6.2. Intraosseous access				
			4.6.3. Central i.v. line				
			4.6.4. Arterial line				
			4.6.5. External pacing				
			4.6.6. Internal pacing				
			4.6.7. Defibrillation				
			4.6.8. Cardioversion				
			4.6.9. Volume replacement therapy (infusions) administered		Check box	Record if intention is to increase circulating volume. Do not record if intention is to “keep-line-open”	
				4.6.9.1. Colloids			
				4.6.9.2. Crystalloids			
				4.6.9.3. Blood products			
			4.6.10. Blood products administered	4.6.10.1. Whole blood	Check box		
				4.6.10.2. PRBC			
				4.6.10.3. Liquid plasma /fresh frozen plasma			
				4.6.10.4. Lyoplas			
				4.6.10.5. Other			
		Continuous	4.6.11. Amount of fluid administered		Number	Millilitres given by p-EMS	
		Categorical	4.6.12. Haemostatic dressing	4.6.12.1. Pressure bandage	Check box		
				4.6.12.2. Packing of wound			
				4.6.12.3. Tourniquet			
				4.6.12.4. Pelvic binder			
			4.6.13. Pericardiocentesis				
			4.6.14. Manual chest compressions				
			4.6.15. Mechanical chest compressions				
			4.6.16. Thoracotomy	4.6.16.1. Lateral			
				4.6.16.2. Clamshell			
			4.6.17. EVR			REBOA or other type of EVR	
			4.6.18. IABP				
			4.6.19. Other				
			4.6.20. None				
**4.7.**	Disability- related procedures	Categorical	4.7.1. Fracture reduction		Check box	Procedures performed by p-EMS	For each mission
			4.7.2. Fracture splinting				
			4.7.3. Spinal immobilization				
			4.7.4. Spinal protection				
			4.7.5. Therapeutic hypothermia				
			4.7.6. Thermal protection				
			4.7.7. Amputation				
			4.7.8. Other				
**4.8.**	Other procedures	Categorical	4.8.1. General anaesthesia		Check box	Procedures performed by p-EMS	For each mission
			4.8.2. Sedation				
			4.8.3. Regional anaesthesia				
			4.8.4. Incubator				
			4.8.5. NO given				
			4.8.6. ECMO				
			4.8.7. Resuscitative caesarean delivery/perimortem hysterotomy				
			4.8.8. Other				
			4.8.9. None				
**4.9.**	Medications administered	Categorical	4.9.1. Opioids		Check box	Type of medication administered by p-EMS	For each mission
			4.9.2. Analgesics except opioids				
			4.9.3. Anaesthetics				
			4.9.4. Antiarrhythmics				
			4.9.5. Antibiotics				
			4.9.6. Antidotes				
			4.9.7. Antiemetics				
			4.9.8. Antiepileptic				
			4.9.9. Antihypertensive				
			4.9.10. Bronchodilators				
			4.9.11. Diuretic				
			4.9.12. Electrolytes				
			4.9.13. Fluids (not for keep-line open)				
			4.9.14. NMBA				
			4.9.15. Procoagulant				
			4.9.16. Fibrinolytic				
			4.9.17. Sedatives				
			4.9.18. Steroids				
			4.9.19. Thrombolytics				
			4.9.20. Vasoactive				
			4.9.21. Tranexamic acid				
			4.9.22. Other				
			4.9.23. None				
**4.10.**	Hospital pre-alert done	Categorical	4.10.1. Yes		Bullet list	Physician has informed receiving hospital of patient state before arriving at the emergency room	For each mission
			4.10.2. No				

*SpO2* Peripheral capillary oxygen saturation, *EtCO2* End-tidal carbon dioxide, *ECG* Electrocardiogram FAST- Focused assessment with sonography for trauma, *p-EMS* Physician-staffed emergency medical services, *POC* Point of care, *NMBA* Neuromuscular blocking agent, *ETI* Endotracheal Intubation, *SAD* Supraglottic airway device, *NIV* Non-invasive ventilation, *FiO2* Fraction of inspired oxygen, *PEEP* Positive end-expiratory pressure, *i.v* intra venous, *PRBC* Packed red blood cells, *REBOA* Resuscitative endovascular balloon occlusion of the aorta, *EVR* Endovascular resuscitation, *IABP* Intra-aortic balloon pump, *NO* Nitric oxide, *ECMO* Extracorporeal membrane oxygenation

**Table 5:** Mission outcome and quality indicators

Data variable number	Main data variable name	Type of data	Data variable categories or values	Data variable sub-categories or values	Type	Definition of data variable	How often should variable be reported
**5. Mission outcome and quality indicators**							
**5.1.**	Mission outcome	Categorical	5.1.1. Patient left at scene		Check box	Patient left by p-EMS at scene. If necessary, taken to GP or other	For each mission
			5.1.2. Patient taken to hospital, not escorted by p-EMS			To hospital by EMS or other	
			5.1.3. Patient taken to hospital, escorted by p-EMS				
			5.1.4. Patient declared dead on arrival at hospital				
			5.1.5. Patient declared dead at scene				
			5.1.6. Discharged alive from scene			Patient is alive when leaving scene	
			5.1.7. Transported to hospital in cardiac arrest with ongoing CPR				
			5.1.8. Patient alive at handover			Patient is alive when p-EMS hand over patient to hospital/GP/EMS unit or other	
			5.1.9. Patient alive at discharge from hospital				
			5.1.10. Patient alive at 30 days				
**5.2.**	Was the patient’s “post-p-EMS” followed up and registered?	Categorical			Bullet list	30-day outcome or outcome at discharge from hospital	For each mission
			5.2.1. Yes				
			5.2.2. No				
			5.2.3. Unknown				
**5.3.**	Intubation success	Categorical	5.3.1. Yes, on first attempt		Bullet list	Successful ETI by p-EMS	For each mission
			5.3.2. Yes, after two or more attempts				
			5.3.3. No				
**5.4.**	Complications to ETI	Categorical	5.4.1. Yes	5.4.1.1. SpO2 < 90% (at any time)	Check box		For each mission
				5.4.1.2. Blood pressure below 90 (at any time)			
				5.4.1.3. If TBI: Blood pressure below 120 (at any time)			
				5.4.1.4. Blood pressure above 200 (at any time)			
				5.4.1. 5. Cardiac arrest or severe, clinically significant bradycardia in relation to the procedure			
			5.4.2. No			No complications to ETI	
**5.5.**	Patient with MI	Categorical	5.5.1. Transferred to PCI centre?	5.5.1.1. Yes	Bullet list	Patient meets criteria for myocardial infarction	For each mission
				5.5.1.2. No			
			5.5.2. On-scene time		Number		
**5.6.**	Stroke patients	Categorical	5.6.1. Transferred to a stroke centre?	5.6.1.1 Yes	Bullet list	All patients considered as having stroke by p-EMS	For each mission
				5.6.1.2 No			
		Continuous	5.6.2. On-scene time		Number		
**5.7.**	Cardiac arrest patients	Categorical	5.7.1. Did patient achieve ROSC for more than 5 min	5.7.1.1. Yes	Bullet list	Patients with cardiac arrest	For each mission
				5.7.1.2. No			
		Categorical	5.7.2. If ROSC: patient transferred to a PCI centre?	5.7.2.1. Yes	Bullet list		
				5.7.2.2. No			
**5.8.**	Pain	Categorical	5.8.1. Was the patient’s pain VAS score reduced below 4?	5.8.1.1. Yes	Bullet list		For each mission
				5.8.1.2. No			
				5.8.1.3. Unknown			
			5.8.2. Did the prehospital treatment reduce pain or otherwise control/improve the subjective symptoms and well-being?	5.8.2.1. Yes		As defined by attending p-EMS	
				5.8.2.2. No			
				5.8.2.3. Unknown			
**5.9.**	Did the prehospital interventions improve or stabilize the vital functions?	Categorical	5.9.1. Yes		Bullet list	As defined by attending p-EMS	For each mission
			5.9.2. No				
			5.9.3. Unknown				
**5.10.**	Adverse events during mission	Categorical	5.10.1. Adverse operational events		Check box	Missing material or teamwork issues during mission	For each mission
			5.10.2. Adverse medical events			Any adverse medical events during mission	

*EMS* Emergency medical services, *p-EMS* physician-staffed emergency medical services, *GP* General practitioner,

*CPR* Cardiopulmonary resuscitation, *ETI* Endotracheal intubation, *SpO2* Peripheral capillary oxygen saturation, *TBI* Traumatic brain injury, *PCI* Percutaneous coronary intervention, *MI* Myocardial infarction, *ROSC* Return of spontaneous circulation, *VAS* Visual analogue scale

### Round IV

The included variables were presented to the experts. After feedback from the experts the wording of variables 1.23.6 and 3.5.6. were changed from “Chest pain, excluding MI” to “Chest pain, MI not confirmed”. Variable 3.8.4. “Systolic blood pressure (SBP) not recordable” and 3.10.4. “SpO2 not recordable” were added. Variables 3.13.1. and 3.13.2. were changed to record the VAS score instead of pain as none, moderate or severe and variable 4.6.17. was changed from “Resuscitative endovascular balloon occlusion of the aorta (REBOA)” to “Endovascular Resuscitation (EVR)”.

### Round V

The experts approved the final version of the template (Table 1, 2, 3, 4 and 5).

## Discussion

### Main findings

Using Delphi methodology, we have updated a template for standard documentation in p-EMS. The new dataset includes new data variables and the template was expanded from 45 to 73 main variables.

### Fixed system variables

Throughout the world, there are large differences between p-EMS [21–23], and fixed system variables are important to analyse any influence of system factors and compare systems [11, 24]. The experts suggested reporting all fixed system variables annually. Furthermore, the experts chose to include two variables related to quality. The reason for including these data in this section is that they describe the quality of the system rather than the quality delivered during each mission.

### Event operational descriptors

There is no consensus in the literature on how to report mission times [15, 25, 26] and the experts had several suggestions, i.e., exact times (hh:mm), time intervals (dispatch time, on-scene time, etc.) and time reported as year/month/day/hour of event. Response time (time from unit is dispatched to at patient side), on-scene time and transport time (from patient leaving the scene to arrival at the hospital) and time from alarm to arrival at the hospital are all reported in various templates. We argue that by reporting exact times, all desired time intervals can easily be calculated; therefore, exact times should be documented.

The time of the event is usually not possible to accurately identify. In trauma, the time of the event will be distinct, but for other diagnoses a clearly defined start time is often missing. The time when a call is received at the emergency medical communication centre (EMCC) is a distinct time that is easy to document, substituting for the time of the event. This was also emphasized by the experts.

P-EMS differ in service profile, and documenting dispatch type is important for benchmarking. Some services are dispatched to all types of emergency missions, whereas others are dispatched to specific types, e.g., trauma. Some services have an extensive workload due to consultation responsibilities and medical direction for ordinary EMS. This may affect availability if work hours are restricted.

### Patient descriptors.

Comorbidity is an important risk adjustment measure, but there is no consensus on comorbidity reporting. The original template for reporting in p-EMS used the American Society of Anesthesiologists Physical Status (ASA-PS) scale in a dichotomized form. However, using full ASA-PS scale has been found to be feasible in p-EMS [27], and it is recommended by the experts.

Reporting the present medical problem is crucial for benchmarking. P-EMS have traditionally reported symptoms, but point-of-care diagnostical options are increasingly available, allowing more precise pre-hospital diagnoses [28–30].

The experts recommended reporting physiological data at two different time points: at arrival of the p-EMS and at hand-over or the end of patient care. This corresponds with the original template. Reporting data at two different time points allows for monitoring changes in the patient state and may serve as a surrogate measure for p-EMS performance [31]. For SBP and SpO2, the experts also suggest reporting the lowest value measured. Hypotension is an independent predictor of mortality for traumatic brain injury (TBI) patients [32], and reporting the lowest SBP value will capture hypotensive episodes. Further, automated data capture from monitors are increasingly available, enabling continuous measurement of physiological variables. Continuous reporting may capture dynamic changes in patient state, thereby increasing the precision of p-EMS research.

Pain is frequent in the p-EMS patient population, and pain relief is considered good clinical practice [33]. The original template used a three-part scale for reporting pain while the expert group of the revised template suggest reporting pain according to the Visual Analogue Scale (VAS) [34].

### Process mapping variables

The resulting physiological effects of p-EMS treatment and its relation to outcome remains largely unknown in pre-hospital critical care. Such changes in physiology have earlier been difficult to capture but doing so is now more feasible due to technological developments. The experts emphasized this, and as such an expansion of the process mapping section was suggested.

### Mission outcome and quality indicators

To date, there is no agreement on standard quality indicators in p-EMS but Haugland et al. recently developed a set of quality indicators for p-EMS [35]. Several of these indicators are documented in the revised template but under various sections. Additionally, the experts suggested several other context-specific quality variables related to the individual patient, but these are yet to be validated.

The experts recommend an event-specific long-term outcome measure to be included on a regular basis. The feasibility of capturing this variable as part of a standardized documentation in the p-EMS population remains to be determined.

### General discussion

Several consensus-based templates for reporting in EMS and p-EMS have been created (e.g., trauma, airway handling and cardiac arrest) [14, 15, 26, 36], and studies have proven that data collection according to such templates are feasible [12, 16, 37]. However, to increase the relevance of templates, variables should be coordinated. Of 26 variables in the template on quality indicators in p-EMS [35], five are identical to variables in the current template, six can easily be calculated and three are partially similar. Thus, little extra effort is required to document according to both templates. We believe that the coordination of variables and linking of templates will add value by reducing workload and increasing data capture, thereby facilitating future p-EMS research.

P-EMS are constantly developing, with new diagnostic and therapeutic options available, e.g. pre-hospital blood products, Tranexamic acid, extracorporeal membrane oxygenation (ECMO), thoracotomy and endovascular resuscitation on-scene. To capture these important trends, templates need to be updated regularly. Additionally, the variables shown to be not feasible to document should either be changed or removed. Physiological variables are often reported to be the most often missing variables [38, 39]. In the original template we found the feasibility of collecting physiological data to be good [16], and these variables were not substantially changed in the updated template. Thus, we expect feasibility to be good for physiological variables in the updated template as well.

To be able to compare outcomes, data must be unambiguously defined [26]. A data dictionary with precise definitions will be created for the present template. Furthermore, when implementing the template, it is important to ensure that all requested data are collected. Each service is free to choose whatever supplementary variables it wants, but all core variables should be captured by default, thereby facilitating future research.

Physician-staffed services are more expensive compared to ordinary EMS services making it a limited resource. This emphasize our obligation to use the service for the right patients. Therefore, we continually should strive to identify patients where p-EMS has an additional effect.

To provide a tool for collection of high-quality data is only a first step towards the improvement of p-EMS research. The next step is implementation, which is pivotal for template success. Aiming to increase awareness of the template, we invited experts from all over Europe to participate in its development. We believe this may facilitate implementation. Furthermore, to increase the implementation rate of the template, targeted efforts, such as involvement of stakeholders and highlighting the possibilities which lies within template data research, must be initiated.

Registries (e.g. for trauma and cardiac arrest) have facilitated a large amount of research [14, 40, 41]. In p-EMS there is currently no joint register and each national service manages its own data. Furthermore, data are often registered on paper and later converted to digital format. Automated data capture from monitors and updated digitized data catchment tools could allow for complete template data to be imported directly into a common registry. This would provide a substantial opportunity for joint research. If such a registry could also link template data to outcomes and standardized coding systems for process and outcome issues, we may be able to assess e.g. for which patients p-EMS are useful, which procedures should be performed out-of-hospital and which procedures should not. However, the ethical and legal requirements of data sharing for research purposes (e.g. General Data Protection Regulation (GDPR)) must be taken into account and a substantial work to adhere to the current regulations are needed to succeed.

In the present study, we applied a Delphi method. This approach is in contrast with the Nominal Group Technique (NGT) that was used in the development of the original template. The classic Delphi method applies questionnaires with e-mails whereas the NGT involves a physical meeting with experts to reach a consensus [42]. The methods can also be combined into a modified NGT that starts with a Delphi process and ends with a physical meeting as a final step before consensus. Because this is an update of an existing template, we considered a physical meeting to be unnecessary. Furthermore, we wanted to ensure anonymity of the experts to prevent authors from favouring certain responses.

Reaching agreement is fundamental in Delphi studies, but a commonly accepted definition of consensus is absent [43]. In the present study we defined consensus as variables rated ≥4 (on a scale from 1 to 5) by > 70% of experts. We consider this a transparent and systematic method for reaching a consensus.

## Limitations

The recruitment of experts is prone to selection bias. For recruitment we used a set of predefined criteria and recruited experts from the EUPHOREA network consisting of representatives from p-EMS throughout central Europe, UK and Scandinavia. The low number of participants (9–11 physicians) may have introduced a selection bias. However, we managed to recruit a representative cohort of p-EMS physicians representing a broad range of European p-EMS. The physician-staffed services represented in the expert group are amongst the most active services in Europe and we believe this ensures generalizability of the results and that the effect of potential selection bias is minimized. By keeping proposals anonymous, we have avoided the effect of favouring proposals from certain experts.

## Conclusions

Using a Delphi method, we have updated and revised the template for reporting in p-EMS. We recommend implementing the dataset for standard reporting in p-EMS.

## Data Availability

The datasets used and/or analysed during the current study are available from the corresponding author on reasonable request.

## Abbreviations

AF: Atrial fibrillation
AFL: Atrial flutter
ASA-PS: The American Society of Anesthesiologists Physical Status
AV-block: Atrioventricular block
CO2: Carbon dioxide
CPR: Cardiopulmonary resuscitation
DASH1a: Definitive airway sans hypoxia/hypotension on first attempt
ECG: Electrocardiogram
ECMO: Extracorporeal membrane oxygenation
EMCC: Emergency medical communication centre
EMS: Emergency medical services
EtCO2: End-tidal carbon dioxide
ETI: Endotracheal Intubation
EUPHOREA: The European Prehospital Research Alliance
EVR: Endovascular resuscitation
FAST: Focused assessment with sonography for trauma
FiO2: Fraction of inspired oxygen
GCS: Glasgow coma score
GP: General practitioner
I.v.: Intra venous
IABP: Intra-aortic balloon pump
MI: Myocardial infarction
NAAF: Norwegian Air Ambulance Foundation
NACA score: National Advisory Committee for Aeronautics score
NGT: Nominal group technique
NIV: Non-invasive ventilation
NMBA: Neuromuscular blocking agent
NO: Nitric oxide
PCI: Percutaneous coronary intervention
PEA: Pulseless electrical activity
PEEP: Positive end-expiratory pressure
P-EMS: Physician-staffed emergency medical services
POC: Point of care
PRBC: Packed red blood cells
REBOA: Resuscitative endovascular balloon occlusion of the aorta
ROSC: Return of spontaneous circulation
SAD: Supraglottic airway device
SaO2: Arterial oxygen saturation
SAR: Search and rescue
SBP: Systolic blood pressure
SpO2: Peripheral capillary oxygen saturation
SRQR: the Standards for Reporting Qualitative Research
SVES: Supraventricular extrasystole
TBI: Traumatic brain injury
VAS: Visual analogue scale
VESmono: Ventricular extrasystole, monomorphic
VESpoly: Ventricular extrasystole, polymorphic
VF: Ventricular fibrillation
VT: Ventricular tachycardia
